# Locked lung by looped hernia: a case report

**DOI:** 10.1186/1757-1626-2-29

**Published:** 2009-01-08

**Authors:** Abu-Ahmed Z Rahman, Mukta Panda

**Affiliations:** 1 Department of Internal Medicine; UTCOM, Chattanooga, 960 East Third Street Suite 200, White Hall Building, Chattanooga, TN 37403, USA; 2 UT College of Medicine, Chattanooga, 960 East Third Street Suite 200, White Hall Building, Chattanooga, TN 37403, USA

## Abstract

**Background:**

Large pleural effusions are usually symptomatic. We report a patient with asymptomatic massive left sided pleural effusion with left lung collapse secondary to a traumatic diaphragmatic hernia.

**Case presentation:**

A 44 year old male presented with recurrent pleural effusions over six weeks. His pleural effusion was first diagnosed incidentally on a chest X-ray after a fall. Extensive diagnostic studies were unyielding for the etiology of the effusion. A diagnostic and therapeutic video assisted thoracoscopy revealed a diaphragmatic hernia with inflamed, incarcerated omentum. After hernia repair there was no recurrence.

**Conclusion:**

This case underscores the obscure presentation of an incarcerated diaphragmatic hernia presenting as massive recurrent pleural effusions.

## Background

Pleural effusion is commonly encountered in clinical practice. It can cause various respiratory symptoms, constitutional symptoms and symptoms related to underlying disease process. Though some patients may be asymptomatic with a small pleural effusion, most are symptomatic and often in significant respiratory distress, especially when the effusion is massive. Diaphragmatic hernia is an uncommon cause of pleural effusion. No incidence data are available but multiple cases are reported [1,3-5]. In most of the reported cases the diaphragmatic hernia was trauma induced.

## Case presentation

**Figure 1:**
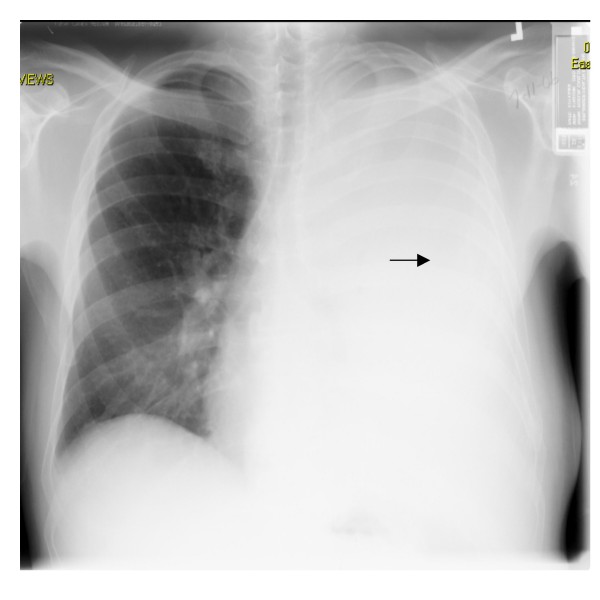
**Chest X-ray (PA view): Re-accumulation of massive left sided pleural effusion (arrow)**.

**Figure 2:**
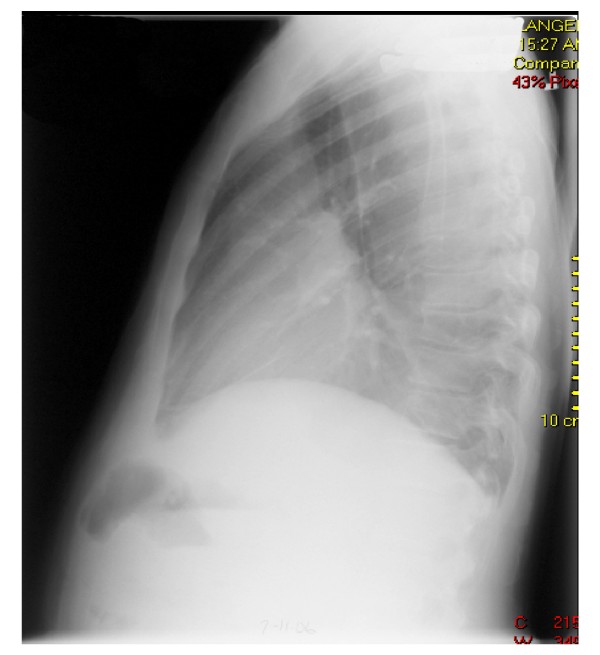
**Chest X-ray (lateral view): Re-accumulation of massive left sided pleural effusion**.

**Figure 3:**
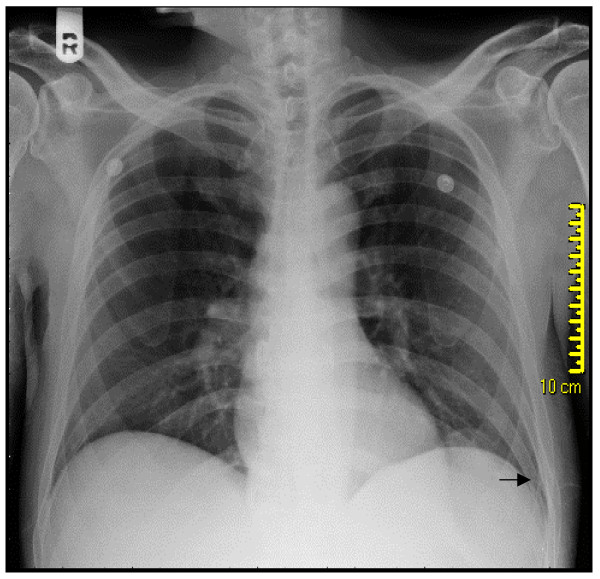
**Initial chest X-ray: fracture of left 8th, 9th and 10th ribs (arrow)**.

A 44 year old Caucasian male was referred by his primary care physician for a recurrent asymptomatic left sided massive pleural effusion. The pleural effusion was first discovered on a chest X-ray obtained to follow-up his left 8^th^, 9^th ^and 10^th ^rib fractures from an accidental fall. The effusion was recurrent over one and a half month even after drainage with thoracentesis and chest tube placement (Fig. 1 &2). The initial chest X-ray immediately after his fall that revealed the acute fractures did not show any pleural effusion or blunting of costophrenic angles (Fig. 3). The patient had no significant past medical history, denied recent travel, but admitted to smoking tobacco and consuming alcohol. The patient worked, as a maintenance man, but at the time of presentation was unemployed.

**Figure 4:**
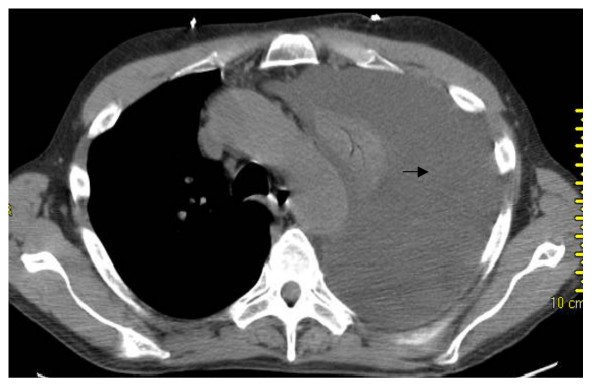
**CT chest: massive pleural effusion left side (arrow)**.

**Figure 5:**
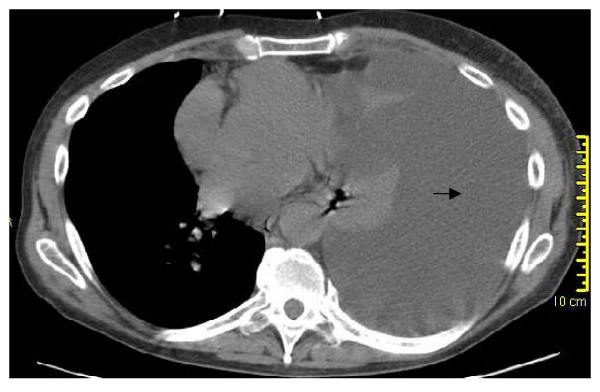
**CT chest: massive pleural effusion on left side (arrow)**.

**Figure 6:**
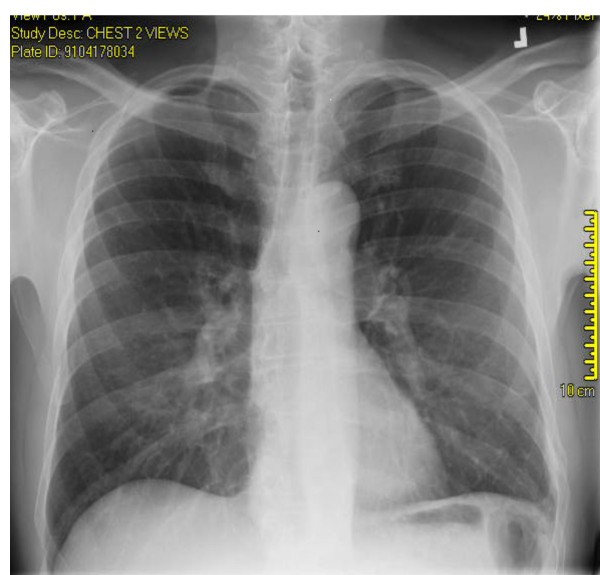
**Chest X-ray at follow up: 5 months after repair of diaphragmatic hernia**.

At presentation the patient had normal vital signs with an oxygen saturation of 93% on room air. Physical examination revealed an average built man in no apparent distress, non-labored breathing, central trachea, absent breath sounds with a stony dull percussion note on the left hemi thorax. Abdomen was soft and non tender with a palpable liver 10 cm below the costal margin. Laboratory investigation revealed mild anemia, hypoalbuminemia (3.1 gm/dL), normal BUN, creatinine, electrolytes and urine analysis. Arterial blood gas (ABG) analysis showed pH 7.48, PaCO_2 _34 mm Hg, PaO_2 _63 mm Hg, HCO_3 _25 mEq/L, oxygen saturation 93% on room air. Pleural fluid study showed lactate dehydrogenase (LDH) 112 U/L, pH 7.80, WBC 76, Polymorphs 40%, Eosinophil 1%, glucose 139 mg/dL, albumin 0.90 gm/dL, RBC 1300/mm^3^, amylase 169 U/L, pleural fluid – serum LDH ratio was 0.48. Pleural fluid gram stain, cultures (aerobic, anaerobic, AFB, fungal) and cytology were all negative. Computerized tomography of chest showed the massive left-sided unilateral pleural effusion with complete collapse of the left upper and lower lobes, but no pulmonary embolism or malignancy. (Fig. 4 &5). Echocardiogram was normal. Ultrasound of abdomen was remarkable for fatty liver, but no ascites. Magnetic resonance cholangio-pancreatography (MRCP) showed normal pancreas and biliary system without ascites. Tuberculin skin test, ANA, ANCA, AMA, ceruloplasmin, TSH, HIV, RPR, and Hepatitis virus serology were normal. Due to frequent massive re-accumulation a diagnostic and therapeutic video assisted thoracoscopy (VATS) procedure was performed, which revealed a 2 cm diaphragmatic hernia with inflamed, friable, incarcerated omentum and small amount of bowel. This inflamed omentum was determined to be the etiology for the recurrent pleural effusion. About 3.5 liters of pleural fluid was removed by VATS. Histopathology was consistent with moderately inflamed omentum. Hernia was repaired and talc-pleurodesis was performed. There was no re-accumulation on follow-up over 2 years (Fig. 6).

## Discussion

Our patient had an intriguing presentation of massive pleural effusion with underlying lung collapse without significant symptoms. A possible explanation for the asymptomatic presentation can be the sedentary lifestyle and good pulmonary reserve of the right lung. Establishing an etiology of this pleural effusion was puzzling. While his non-toxic clinical presentation, normal leukocyte count and pleural fluid findings were consistent with transudative effusion, the unilateral distribution of the pleural effusion was inconsistent. CT imaging of chest did not identify the diaphragmatic hernia, which may be due to small size of the hernia. Usually diaphragmatic hernia does not cause pleural effusion unless it contains incarcerated viscera [1,2,6]. Almost all massive pleural effusions with diaphragmatic hernia contained a strangulated omentum [1,6]. Schreier et al reported a case of hemorrhagic pleural effusion from infarcted omentum in a non-traumatic diaphragmatic hernia [4]. Several cases were reported on World War II and Vietnam veterans [1,5].

Presenting symptoms are much varied, but usually pain from trauma if recent, respiratory distress, gastrointestinal symptoms and abdominal pain from intrathoracic strangulation of abdominal organ [5]. The time interval between the onset of traumatic diaphragmatic hernia and the presentation with symptom may vary from days to years. Presentation as late as 26 years after the original accident are also reported [3,6]. In our case the effusion developed within a week. Initial thoracentesis usually yields serosanguinous fluid especially at an early stage. A unilateral pleural effusion especially one on left side, with above clinical presentation and serosanguinous pleural fluid should make one highly suspicious for a strangulated diaphragmatic hernia. If no recent trauma is reported, trauma in the past may be the clue. A CT scan of the chest may be performed early to evaluate a hernia and to assess other possible diagnoses such as malignancy, pulmonary embolism or esophageal rupture. Pleural fluid drainage with repair of the diaphragmatic hernia is the definitive treatment.

## Conclusion

Pleural effusion from incarcerated diaphragmatic hernia is an infrequent diagnosis. It is less often entertained as a differential diagnoses in the work up of a pleural effusion. Pathology is often obscure with a potential to miss, causing the patient recurrent morbidity and even the risk of death. Early diagnosis of this potentially curable pathology will significantly reduce patient suffering and economic burden.

## Abbreviations

Fig.: Figure; BUN: Blood Urea Nitrogen; PaCO_2_: Partial pressure of arterial Carbon dioxide; PaO_2_: Partial pressure of arterial Oxygen; HCO_3_: Bicarbonate; AFB: Acid Fast Bacilli; ANA: Anti Nuclear Antibody; ANCA: Anti Nuclear Cytoplasmic Antibody; AMA: Anti Mitochondrial Antibody; TSH: Thyroid Stimulating Hormone; HIV: Human Immunodeficiency Virus; RPR: Rapid Plasma Reagin; CT: Computed Tomography; WBC: White Blood Cell; mEq: Milli Equivalent; U/L: Unit/Litre.

## Consent

The patient expired several years later from accidental poisoning. Written informed consent was obtained from the patient's mother for publication of this case report and accompanying images. A copy of the written consent is available for review by the Editor-in-Chief of this journal.

## Competing interests

The authors declare that they have no competing interests.

## Authors' contributions

The manuscript was prepared by the AAR, with guidance, input and critical review obtained from MP. All authors read and approved the final manuscript.
